# Exploring routine palliative care delivery to inform tailored digitalization approaches - a qualitative interview study

**DOI:** 10.1186/s12904-026-02345-0

**Published:** 2026-10-16

**Authors:** Alina Weisser, Natalie Öhl, Ann-Kathrin Mahlein, Christoph Ostgathe, Tobias Steigleder, Maria Heckel

**Affiliations:** Department of Palliative Medicine, University Hospital Erlangen, Friedrich- Alexander-Universität Erlangen-Nürnberg, Werner von Siemens Straße 34, Erlangen, 91057 Germany

**Keywords:** Palliative care, Home care services, Social change, Digital health, Qualitative research

## Abstract

**Background:**

Digital technologies are increasingly being introduced into palliative care. However, little is known about the implementation of new technologies from the perspectives of healthcare professionals and informal caregivers. This study explores current specialist palliative home care, particularly the circumstances of the actors as well as information exchange and care processes, to inform the implementation of new technologies.

**Methods:**

A qualitative study involving problem-centred interviews was conducted with healthcare professionals and informal caregivers involved in specialist palliative home care in Germany. The Interviews were analysed by structuring qualitative content analysis guided by the CARE-HOUSE Framework, with inductively developed subcategories.

**Results:**

Eleven interviews were conducted with eight healthcare professionals and three informal caregivers. Participants described specialist palliative home care as holistic and well-functioning but characterized by complex and fragmented communication pathways. A wide range of actors, characteristics and means and content of current data transfer could be collected. Informal caregivers frequently act as intermediaries in information exchange, resulting in a considerable organizational burden. The participants identified challenges in current care practice related to communication, data management and institutional barriers. Both groups identified centralized digital information exchange as a major opportunity to address these challenges.

**Conclusions:**

The findings suggest that digital technologies have the potential to improve communication, coordination, and information exchange in specialist palliative home care while reducing administrative burden. Successful implementation requires adaptation to palliative care, providing a user-centred design that preserves the central role of personal interaction in palliative care and accounts for the diverse needs, competencies, and roles of healthcare professionals, informal caregivers, and patients.

## Background

Digitalization will have an impact on health care in Germany [1, 2]. Additionally, palliative care is affected by this development [3], as new technologies are expected to shape its future [4]. This is evident in the establishment of working groups dedicated to advancing digitalization in this field [3] and the development of technological approaches designed to support palliative and end-of-life care [5–11]. These include virtual care solutions, the digitalization of established tools, the use of telehealth and the use of digital applications, many of which focus on facilitating home-based care, as home remains a preferred place of death in Germany [5, 6, 8, 12, 13].

The TEAM-X project follows the same objective by developing a digital application to support patients in specialist palliative home care (Spezialisierte Ambulante Palliativversorgung, SAPV), a German interdisciplinary model that delivers comprehensive palliative care in patients’ homes through multidisciplinary teams for those with complex needs [14, 15]. It focuses on health status monitoring, symptom assessment, and data exchange. In home-based care, digital solutions may improve access to care by addressing challenges related to distance and time [10, 16–18]. Before such a system can be implemented, it is crucial to identify key areas in the field where technology can provide support in a meaningful manner. In a second step, the implementation of the system should be evaluated from the perspectives of relevant stakeholders to ensure that its further development is aligned with the realities, needs, and structural conditions of the healthcare system [19–22]. Given the vulnerability of patients and their informal caregivers, palliative care is particularly prone to ethical and social challenges [23]. However, research on the implementation of systems such as TEAM-X remains limited, especially with respect to the perspectives of both healthcare professionals (HCPs) and informal caregivers (ICs) [16]. Healthcare professionals’ roles are expected to drastically change due to the introduction of such new technologies aiming to facilitate communication and data exchange, which require a combination of technical knowledge, management abilities, soft skills, and communication skills [24]. Informal caregivers also play a critical role in palliative care and should therefore be considered key actors when evaluating interventions that impact care practices [25–28].

The present study examines specialist palliative home care using the TEAM-X system as an example. The in-depth analysis of current routine palliative care from the perspective of healthcare professionals and informal caregivers will allow digitalization, in a more targeted manner, considering the circumstances of the actors, their roles, and the challenges and benefits of information exchange in palliative routine care.

## Methods

This study is one of two substudies within a broader interview-based exploration of the social implications of TEAM-X [22]. Both substudies were based on the same sample and data collection procedures but addressed different research questions and analytical focuses. A detailed description of the setting, study design, sampling, recruitment and data collection procedures is provided in the companion substudy [22]. The study was approved by the Ethics Committee of the Medical Faculty in Erlangen on January 9, 2023 (22-407-B).

### Setting and study design

The study is rooted in constructivist theory [29]. Data were collected through semistructured problem-centred interviews [30] and analysed via qualitative content analysis [22, 31]. 

### Sampling

The sample was selected on the basis of convenience and included adults (≥ 18 years) who were either part of a specialist palliative homecare team or had at least four weeks of experience as informal caregivers in this setting. The exclusion criteria were lack of consent, inability to provide consent, or insufficient language skills. To achieve data saturation [32–34], recruitment aimed to include 6–8 healthcare professionals and 6–8 informal caregivers. Palliative care patients were not recruited at this stage because the expected interview length was considered too burdensome. The participants received a 50 Euro bookstore voucher. For brevity and clarity, all specialist palliative home care team members including administrative staff are referred to as healthcare professionals [22]. 

### Recruitment

Recruitment was conducted between January and May 2023 through specialized palliative care teams known to the authors, professional associations (Association for Specialist Palliative Home Care in Bavaria), a congress (German Association for Palliative Medicine, DGP), hospice organizations, social media, the department’s Patient and Public Involvement Initiative (PPI), and snowball sampling.

### Data collection

The interviews were audio-recorded via Zoom Video Communications and conducted by a research fellow (AW, psychologist) after specialized interview training, who had no prior contact with the participants. Slides illustrating the TEAM-X ecosystem and care scenarios—covering palliative care needs, symptom monitoring, opioid prescription, and advance care planning—were used to guide discussions. The development of the interview guide was guided by the CARE-HOUSE Framework [35] and explored data exchange in palliative care, the impact of new digital health technologies, and participants’ perspectives. The framework conceptualizes the social implications of digital health technologies in palliative care, including their potential impact on actors’ roles and characteristics, interactions, tasks and processes, and broader care context [35]. A short version of the interview guide can be found in the supplementary section of the companion substudy [22].

### Data analysis

Data saturation saturation was discussed after conducting all interviews. The interviews were transcribed following Pehl and Dresing [36] and analysed by structuring content analysis [29] with MAXQDA 2022/2024 (release 22.0.7/24.0.0) [37]. The transcripts were not returned to the participants but were checked by AW for accuracy. To ensure quality, two researchers (AW and AKM (master’s student, medical process management)) independently coded the data, compared the results, resolved discrepancies, and consulted a third researcher (MH, social worker and nursing scientist) in feedback loops when needed. The coding was iteratively refined. After 6 interviews (55%), MH conducted a consistency check. The MAXQDA logbook and memos document coding rules, modifications, and decisions.

### Coding system

The coding system was based on research questions, the interview guide, and components of the CAREHOUSE Framework [35], with inductive subcategories developed during coding. In Table 1, the codes used for this substudy are shown.

**Table 1:** Content coding system

actors	actors mentioned by participants	
	characteristics	patients
		informal caregivers
		healthcare professionals
	tasks and roles	current care routine
		palliative care needs
		symptom burden and health status
		opioid prescription^1^
		advance care planning^1^
		tasks with the new technology^1^
		roles of informal caregivers
		tasks of informal caregivers
	relations and interactions	
	information transfer	means of information transfer
		content of information transfer
		existing systems
assessment of the current care practice	functioning processes	treatment of patients
		communication, data collection and exchange
		processes^1^
	difficulties and challenges	treatment of patients
		communication, data collection and exchange
		existing care processes do not work (sufficiently)^1^
		informal caregivers are overburdened^2^
		reservations about palliative care^2^
	general wishes	

*Legend*: ^1^: only named by healthcare professionals / ^2^: only named by informal caregivers

Formal codes were used to distinguish between text referring to current and prospective care practice, identify references to proposed scenarios (see “data collection”), and highlight descriptions of expectations for good palliative care. For this substudy, the analysis focused on actors; their characteristics, tasks and roles; and their interactions and information transfer, alongside participants’ general assessments of current care practice.

### Use of generative AI and AI-assisted technologies in the writing process

During the preparation of this manuscript, the authors used ChatGPT, DeepL and Springer Nature’s language editing service (Curie) to improve the language, readability, and overall clarity of the text. All outputs were carefully reviewed and edited by the authors, who take full responsibility for the final content of the manuscript.

## Results

Recruitment and participant characteristics were identical to those of the companion study (see Methods section) [22]. 

### Recruitment

All eight responding healthcare professionals met the eligibility criteria. One out of four informal caregivers was excluded because they had only one week of caregiving experience. While reasons for refusal were not systematically recorded, healthcare professionals frequently mentioned a lack of time or interest. Recruiting informal caregivers with the necessary experience and emotional resilience proved difficult. Despite efforts to recruit 6–8 informal caregivers, only three were enrolled within the recruitment period. Their data were nevertheless included as supplementary findings to capture this perspective, although the small sample size did not allow for direct comparisons with the other participant group. All interviews were completed successfully and required no repetition.

### Participant characteristics

Saturation was reached after eleven interviews were conducted between March and May 2023. The average interview lasted 54:46 min (SD = 7.55). Two participants were male, with an average age of 52 years (SD = 10.4; range: 34–68). Demographic details are provided in Table 2.

**Table 2:** Demographics of healthcare professionals and informal caregivers

Healthcare professionals					
number of participants	age [mean (SD)]	gender (%)	profession (%)		years of experience in palliative care (%)
8	52.25 (10.3)	female (75%)	medical director (25%), nurse (25%), office management (12.5%), nursing director (12.5%), team leader (12.5%), physician (12.5%)		eleven or more years (75%), six to ten years (12.5%), less than one year (12.5%)

Informal caregiver					
number of participants	age [mean (SD)]	gender (%)	relation to patient	distribution of responsibility (%)	duration of care
3	56.67 (12.86)	Female (100%)	granddaughter, daughter, partner	Primarily responsible (66.67%), team of three (33.33%)	two and a half months, one and a half years, two and a half years

### Current specialist palliative home care practice

The participants described palliative care as holistic and compassionate support, characterized by symptom relief, personal connection, respect for patient preferences, and coordinated care structures. Overall, all participants found that the current palliative home care practice was sufficient for the principles of good palliative care. Digitalization was described as already playing a role in current care practice, although participants reported varying experiences with it, ranging from positive to negative.

The participants stressed the collaborative and flexible nature of care provision. Healthcare professionals also highlighted the relevance of psychological and spiritual needs in care decisions. They revealed that personal interaction, rather than specific procedures, is important in palliative care and highlighted the use of personal perception as a treatment tool.

“… because the nurse sees that, she acts accordingly, she has skills that I might not have. And she reaches the patient on a completely different level, which is only possible through personal contact.“ Int 3, HCP

They emphasized the importance of personal contact for assessing and implementing advance care planning, suggesting that this aspect should not be replaced digitally. Similarly, opioid prescribing and medication documentation were considered tasks requiring oversight by a trained healthcare professional rather than being fully digitalized.

### Actors

#### Actors, interactions, tasks and roles

**Fig. 1:**
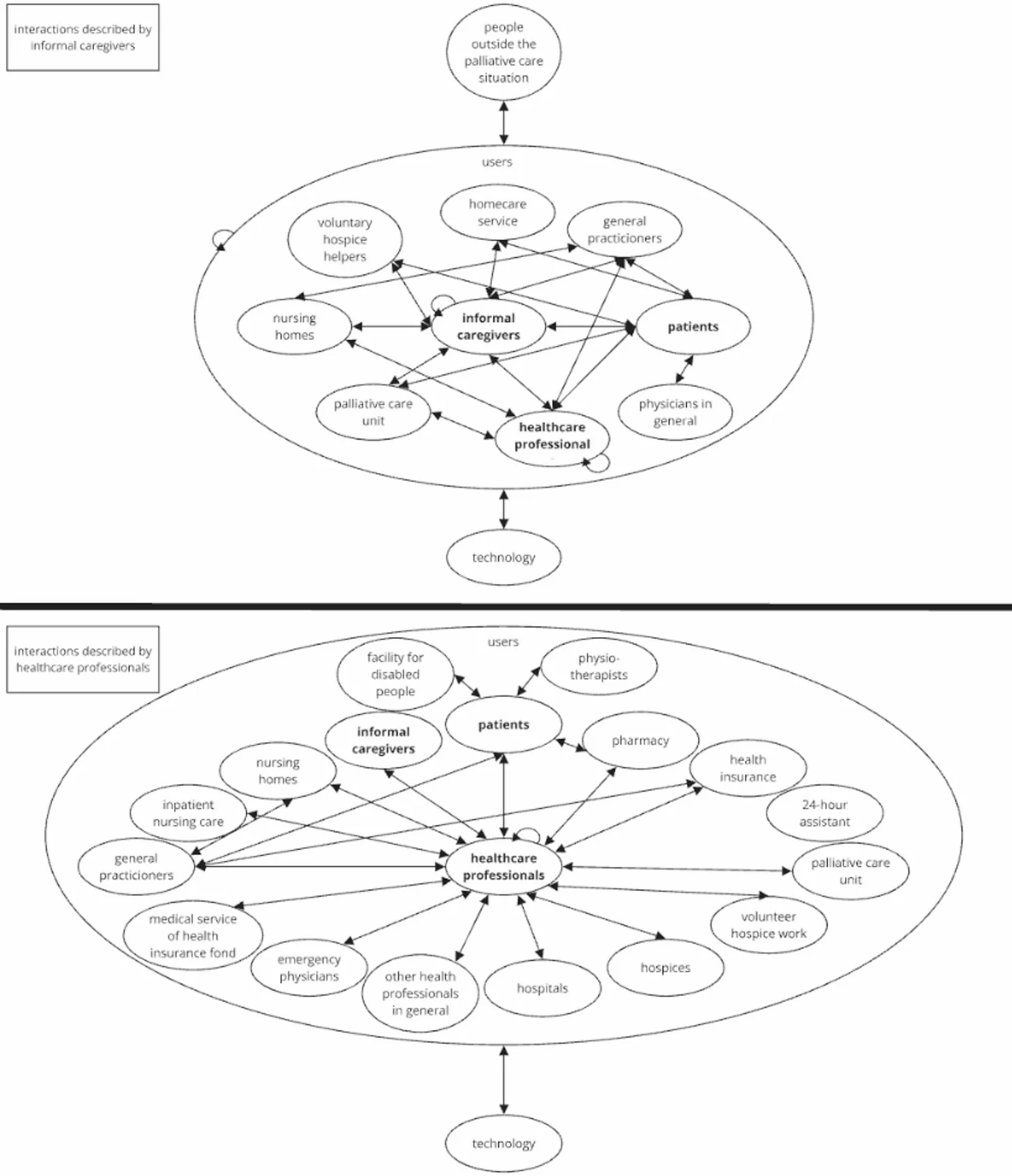
Interactions as described by the participants

A variety of actors and interactions between actors and institutions in care practice are named by both groups (Fig. 1). Healthcare professionals named more interactions between healthcare professionals, whereas informal caregivers frequently described their own and the patient’s interactions with healthcare professionals. If new technology was to be implemented, palliative care associations were suggested as potential promoters.

On the basis of the participants’ statements, no specific tasks or roles could be clearly identified. The role of informal caregivers emerged as the only distinctly described role. They reported being primarily responsible for coordinating the current care situation, communicating the patient’s status to healthcare professionals, and ensuring the implementation of care plans. The participants expected that new communication and data exchange technologies would allow informal caregivers to take on a less active coordinating role and devote more attention to patients’ needs.

#### Actors’ characteristics

The collected characteristics consist of features that the participants mentioned in the context of the named main actors and are summarized in Table 3. Digital competence was less commonly attributed to older individuals, and a significant portion of the patient group tends to be older.

“…well, if I think about our patient population, I can say that they are predominantly elderly to very elderly people.”

**Table 3:** Characteristics associated with actors

Category	Description
Patients	
age	tend to be older, some younger
emotional state	reconciled with their condition, psychologically burdened, suffering, overburdened, helpless, stressed, lonely, fearful, fearing dismissal of complaints, burdened with suffering in conflict situations
cognitive state	some fit, some demented or cognitively impaired
motives	self-determination, high degree of certainty regarding their end-of-life decisions (wanting to end their lives, not wanting to be hospitalized, not wanting life support measures)
physical condition	dying, severely ill, high symptom burden, limited motor skills; in some cases relatively high level of physical functioning
handling of their condition	overwhelmed with the assessment of their condition; different strategies, with some preferring medication and others relying on spirituality, physical movement, being outside
social aspects	socially weak, not wanting visitors
medical skills	some untrained
communication abilities	some communicative, some unable to express themselves
technological skills	varying levels of technological skills, with most patients having low skills
Informal caregivers	
emotional state	proud, resilient, deeply affected, (over)burdened, helpless, in need of reassurance, ashamed
social aspects	impoverished
interaction with patients	supportive, not being able to support, invasive
social aspects	heterogeneous family systems
constraints	not being able to manage all tasks, some people in family systems are not supportive
medical skills	some with medical background, others rather untrained
attitudes towards palliative care	positive view on palliative care
technological skills	varying levels of technological skills
attitudes towards technology	less critical towards new technologies
attitudes towards data security	willing to share data, prioritizing rapid data exchange
Healthcare professionals	
age	old general practitioners, young healthcare professionals in specialist palliative homecare
emotional state	overburdened general practitioners
interaction with patients	open and friendly healthcare professionals in specialist palliative homecare
care delivery challenges	language barriers in nursing care
medical skills	good training in nursing care
palliative care skills	healthcare professionals in specialist palliative homecare guided in their work by their own perception and experience; limited knowledge about palliative care among general practitioners; lack of palliative competence in nursing homes
attitudes towards technology	some averse to technology, some inclined towards technology; resistant to technological change

Int. 6, HCP.

Informal caregivers were described as overburdened, mainly as a result of the mediating role the take on (see “actors, interactions, tasks and roles” or “challenges”) or organizational tasks (e.g., managing collected patient data, see “current data transfer”). Additionally, they highlighted that rapid data exchange was more critical than data security, as it ensured that patients’ autonomy was respected and that their preferences were implemented. Healthcare professionals represented a wide range of ages and attitudes toward technology. Moreover, gaps in palliative care knowledge were identified, particularly among nursing home staff and general practitioners.

### Current data transfer

#### Means and contents

In current practice, data exchange seemed quite complex and extensive, with many differing means and contents (see Table 4). Healthcare professionals referred to rather time-consuming procedures, such as analogue documentation of patient information, then transfer into a digital system, more or less unstructured subjective data collection and exchange and other unstandardized procedures.

“… when we are with the patient, we usually make notes on paper, also according to a scheme, there is an assessment sheet. And then we transfer this to the ISPC when we document the admission or the visit, so to speak, and then it is recorded digitally.”

**Table 4:** Means and content of data transfer

Means and content of data transfer		Described by healthcare professionals	Described by informal caregivers
currently used systems	digital system for palliative care in Germany (ISPC) [mentioned by interviewees 2, 5, 6, 7, 9 and 10, all HCP]	x	
	electronic health system (KAS)	x	
	book for narcotics	x	
	data carrier procedure	x	
	documentation system for prescriptions		
means	telephone	x	x
	general digital exchange	x	x
	in person	x	x
	data exchange via caregivers and patients	x	x
	email	x	
	fax	x	
	post	x	
	paper to scan to digital system	x	
	in paper		
content	medication plan	x	x
	prescription	x	x
	advanced care planning	x	x
	medical letters	x	
	invoices with the health insurance company	x	
	appointments	x	
	personal and medical data	x	
	patient emergency documents	x	
	declaration of consent	x	
	registration for hospice support	x	
	data protection declaration	x	
	contracts	x	
	emergency forms	x	
	application forms for palliative care		x

Int 6, HCP.

Informal caregivers and patients were identified as conduits for information.

“… I first called specialist palliative homecare and made an initial appointment. Then they told me what prescriptions I needed from my general practitioner, what had to be in them and so on. And then I just went to the general practitioner and picked it up, then took it back to the specialist palliative homecare office and that''s how it started, right?”

Int 11, IC.

#### Beneficial current procedures in terms of communication

Healthcare professionals valued several aspects of current procedures: the exchange of narrative data, quick telephone communications, time for patients and trusted relationships with personally known institutional partners. Informal caregivers perceived good communication with everyone in the care system, including the specialized outpatient care team and general practitioners.

#### Challenges of current communication and data transfer procedures

The interviews revealed significant challenges hindering optimal palliative care delivery, as reported by both groups, primarily related to communication, data management, and institutional barriers.

With respect to interprofessional communication, healthcare professionals reported insufficient exchange between care providers, a lack of centralized data storage, and limited access to information from other institutions. Informal caregivers reported poor communication within and between teams, noting a lack of transparency in decision-making. They described difficulties reaching healthcare professionals, time-consuming interactions with general practices, and inefficient communication with specialized outpatient care teams. Informal caregivers also noted insufficient exchange between care providers and wished not to take on a mediating role in data exchange and not being in charge. They advocated for centralized data storage without the need for informal caregivers to act as intermediaries. Triangulated data exchange, e.g., via general practitioners, was considered inefficient and inconvenient.

“… It became very difficult for us from the moment we put her in a nursing home. Because that''s when communication stopped working at all […] because then it''s all about five corners. So at some point we brought in the specialist palliative homecare team and then the communication was always: nursing home, informal caregivers, nursing home, general practitioner, nursing home, specialist palliative homecare team. But the others don''t communicate with each other.”

Int 1, IC.

In terms of data management, both groups reported challenges. Healthcare professionals described excessive reliance on paper-based systems, leading to high documentation effort and lack of structured processes, including standardized methods for tracking symptom burden, side effects, and medication. Informal caregivers, often burdened with managing data exchange, reported struggles with paper-based systems that risked data loss. Both groups emphasized that these practices resulted in suboptimal treatment outcomes, with informal caregivers prioritizing rapid data exchange over data security.

Institutional barriers further complicate care delivery. Healthcare professionals reported resistance from health insurance companies to digitalization efforts and restrictions on direct insurer contact. While important, data privacy measures were reported to hinder everyday care delivery, particularly in requesting health reports. The healthcare system in general was described as regressive, and participants stated that major difficulties in patient care are to be expected in the future (e.g., recognition of palliative care needs, medication prescription and discharge management from clinics). Healthcare professionals had bad experiences with technical devices and new digital solutions (e.g., e-prescription). They also named financial (e.g., staff with expertise is too expensive), staff- (e.g., not enough staff or not enough with expertise), and time-related problems (e.g., faxing and talking via phone) as well as problems accessing possible palliative patients. As a result, healthcare professionals expressed a desire for increased digitalization, improved networking, and centralized data storage to streamline processes. They emphasized the need for something such as a health card that would allow access to patient data prior to medical visits, facilitating more efficient care.

## Discussion

This study analyses how healthcare professionals and informal caregivers experience everyday care in specialist palliative home care, with a focus on current palliative care practices, actors and their characteristics and information exchange to adapt digitalization approaches to the actors and the palliative home care situation.

### Main results

The participants described current specialist palliative home care as largely aligned with palliative care principles, particularly regarding respect for patients’ wishes, a core aspect of palliative care that supports autonomy and dignity [38], and the preference for home-based care [12, 38].

Personal contact and interpersonal relationships were considered essential therapeutic components of palliative care, particularly in addressing psychological and spiritual needs. New communication and data exchange technologies should therefore complement rather than replace interpersonal interactions [22]. Moreover, the literature suggests that digital technologies may alter the self-understanding, roles and responsibilities of patients, caregivers, and healthcare professionals, highlighting the need for further research on these effects [27]. In particular, the suggested shift of healthcare professionals toward educational and system-management roles deserves further investigation, given the existing workload and time constraints.

A diverse range of actors in specialist palliative home care could be identified. This may serve as a useful foundation for the development of digital interventions, helping to ensure that both are tailored to the specific requirements and complexities of palliative care.

Digital competence and attitudes toward technology emerged as key factors for the implementation of new communication and data exchange technologies, reflecting concerns that insufficient digital skills or reservations toward technology among all actors could hinder their successful adoption and use. Older patients were perceived as having limited digital literacy, whereas informal caregivers and healthcare professionals showed varying levels of technological skills and acceptance. Consequently, digital solutions should be intuitive and accompanied by adequate training and support. Beyond these measures, targeted communication strategies may be needed to address anticipated concerns regarding individuals’ ability to use new communication and data exchange technologies. Highlighting existing digital practices in palliative care [16], such as electronic patient documentation, may be useful for fostering trust in and acceptance of further digitalization.

The participants also described challenges when patients were unable to communicate due to advanced disease, dementia, or medication effects, findings that are consistent with previous literature [39–41]. In such situations, new communication and data exchange technologies may facilitate care by enabling the collection of health-related information without relying on verbal communication. Additionally, it could help prevent unnecessary examinations and treatments at the end of life, which may reduce patients’ quality of life [38].

Informal caregivers are central coordinators of care, a role that is associated with substantial emotional, social, and practical burdens [27, 42–44]. The participants suggested that digital technologies could reduce some of these responsibilities by improving information exchange. However, both our study and previous research suggest that not all informal caregivers are willing or able to engage with new technology, which should be taken into account during their development and implementation [28].

The participants reported varying levels of palliative care expertise in healthcare professionals, particularly in nursing homes and among general practitioners, confirming findings from previous studies [45–47]. Given their central role in palliative care [48] and the existence of misconceptions about palliative care in the medical community [49], all healthcare professionals involved in specialist palliative home care should be actively involved in the implementation of new communication and data exchange technologies. The findings also underline the importance of palliative care education, as training has been shown to improve confidence, knowledge, and attitudes toward end-of-life care [46, 49–53], whereas educational gaps have already been identified among medical students [54].

Various communication pathways were identified. May et al. reported that substantially more digital technologies are used in home-based care than in inpatient settings [16]. However, unlike other studies [18, 55], the participants in our study did not mention real-time digital communication tools such as video consultations or messenger services. When further digitalization measures are implemented, careful consideration should be given to the complexity of existing communication pathways, which pathways are already functioning effectively, and which processes can be sustainably replaced or supplemented. In doing so, it is important to recognize that actors may have preferred modes of communication with which they are already familiar and comfortable [55]. Taking these preferences into account may facilitate acceptance and support the successful integration of new digital solutions.

Communication and information exchange were identified as major challenges. Data transfer was described as complex and documentation-intensive, with informal caregivers frequently acting as intermediaries. Since deficiencies in teamwork, excessive documentation and bureaucracy are well-documented challenges in health care, particularly in palliative care [48, 56], digital solutions may offer benefits by streamlining communication, reducing administrative workload [57], and providing a shared information platform for all the actors involved. Such improvements could strengthen coordination and collaboration while freeing time for direct patient care.

### Strengths and limitations of the study

A major strength of this study is its focus on data exchange processes in specialist palliative home care from the perspectives of both healthcare professionals and informal caregivers, a topic that has received limited attention to date. The study benefits from in-depth qualitative data, targeted expertise, systematic analysis and a diverse sample, providing valuable insights into specialist palliative home care. The qualitative approach enabled an in-depth exploration of participants’ experiences and reflections with the interview method, allowing for flexibility, while the inclusion of participants with diverse backgrounds enriched the findings.

Several limitations should be considered. The deductive coding framework may have constrained the analysis, although inductive subcodes were used to capture emerging themes. Recruitment was challenging, resulting in a small sample, particularly among informal caregivers, and patient perspectives were not included. Some interviews with healthcare professionals were conducted while they were at work or under time pressure. Future studies should consider a longer data collection period to facilitate recruitment, increase the sample size, and allow interviews to be conducted under more favourable conditions.

Although participation may have been emotionally demanding for informal caregivers, recruitment took place only after an appropriate period had passed since the loss. However, informal caregivers are often willing to participate in such studies and may even benefit from sharing their experience [28, 58, 59].

Furthermore, the predominance of highly experienced healthcare professionals may limit the transferability of the findings. Potential differences between urban and rural palliative care settings were not explicitly considered in this study, despite suggested regional disparities. However, the interviewees were recruited from both urban and rural regions, and previous research has not identified significant differences in palliative care provision between these settings [60].

Furthermore, the software systems used by healthcare professionals were not systematically assessed. ISPC was explicitly mentioned by six of the eight healthcare professionals, whereas no other system was mentioned. However, participants may have used other systems without mentioning them, including PalliDoc, which is widely used in specialist palliative home care in Germany [61–63]. Thus, it cannot be determined whether the findings are specific to ISPC or transferable to users of other systems. Differences in software functionality may influence users’ experiences and should therefore be considered when assessing the transferability of the findings.

As with all semistructured interviews, interviewer influence cannot be fully excluded. The researcher’s professional background as a psychologist may have shaped the interview process and interpretation of the findings. To minimize bias, interview training was conducted, and the participants were encouraged to express their views openly and critically [22]. 

### What this study adds

This study provides a detailed understanding of current care practices and derives implications for the integration of new communication and data exchange technologies in specialist palliative home care. Identifying the perspectives of key actors highlights factors that should be considered before implementing digital solutions to ensure a good fit with palliative care principles. The findings can inform the user-centred development of future technologies and guide further research on how digital tools can support care processes while being tailored to core elements of palliative care.

## Conclusion

Overall, the study suggests that the successful implementation of new communication and data exchange technologies in specialist palliative home care requires user-friendly solutions that reduce administrative burden, improve communication and information sharing and support patients with communication impairments. Implementation efforts should also consider varying levels of palliative care competence among healthcare professionals, and the diverse needs and capabilities of all the actors involved. Given concerns regarding reimbursement, data security, and other institutional barriers, political and organizational efforts are needed to support the targeted digitalization of palliative care. Nevertheless, the findings indicate a generally positive attitude toward digitalization, provided that new technologies are tailored to the palliative care context and support, rather than undermine, interpersonal care relationships [22].

## Data Availability

The datasets generated and analysed during the current study are not publicly available since data protection information to participants guaranteed their anonymity in any presentation or dissemination of the study’s findings. As the participants´ responses partly contained detailed information on the specialist palliative home care teams, it is not possible to anonymise data completely. The raw data is therefore unavailable. The datasets are available from the corresponding author upon reasonable request.

## Abbreviations

HCP: Healthcare professional
IC: Informal caregiver
